# Anodal tDCS Over the Left Prefrontal Cortex Does Not Cause Clinically Significant Changes in Circulating Metabolites

**DOI:** 10.3389/fpsyt.2020.00403

**Published:** 2020-05-07

**Authors:** Aaron Kortteenniemi, Alfredo Ortega-Alonso, Amir-Homayoun Javadi, Tommi Tolmunen, Toni Ali-Sisto, Tuukka Kotilainen, Jan Wikgren, Leila Karhunen, Vidya Velagapudi, Soili M. Lehto

**Affiliations:** ^1^ Institute of Clinical Medicine, University of Eastern Finland, Kuopio, Finland; ^2^ Department of Psychology and Logopedics, Faculty of Medicine, University of Helsinki, University of Helsinki, Helsinki, Finland; ^3^ Department of Public Health Solutions, National Institute for Health and Welfare, Helsinki, Finland; ^4^ School of Psychology, University of Kent, Canterbury, United Kingdom; ^5^ Department of Experimental Psychology, Institute of Behavioural Neuroscience, University College London, London, United Kingdom; ^6^ School of Rehabilitation, Tehran University of Medical Sciences, Tehran, Iran; ^7^ Institute of Clinical Medicine and Clinical Nutrition, Kuopio University Hospital, Kuopio, Finland; ^8^ Centre for Interdisciplinary Brain Research, Department of Psychology, University of Jyväskylä, Jyväskylä, Finland; ^9^ Institute of Public Health and Clinical Nutrition, University of Eastern Finland, Kuopio, Finland; ^10^ Metabolomics Unit, Institute for Molecular Medicine Finland (FIMM), University of Helsinki, Helsinki, Finland; ^11^ Psychiatry, University of Helsinki and Helsinki University Hospital, Helsinki, Finland

**Keywords:** brain stimulation, transcranial direct current stimulation, tES, metabolism, mass spectrometry

## Abstract

**Background:**

Transcranial direct current stimulation (tDCS), a putative treatment for depression, has been proposed to affect peripheral metabolism. Metabolic products from brain tissue may also cross the blood–brain barrier, reflecting the conditions in the brain. However, there are no previous data regarding the effect of tDCS on circulating metabolites.

**Objective:**

To determine whether five daily sessions of tDCS modulate peripheral metabolites in healthy adult men.

**Methods:**

This double-blind, randomized controlled trial involved 79 healthy males (aged 20–40 years) divided into two groups, one receiving tDCS (2 mA) and the other sham stimulated. The anode was placed over the left dorsolateral prefrontal cortex and the cathode over the corresponding contralateral area. Venous blood samples were obtained before and after the first stimulation session, and after the fifth stimulation session. Serum levels of 102 metabolites were determined by mass spectrometry. The results were analysed with generalised estimating equations corrected for the family-wise error rate. In addition, we performed power calculations estimating sample sizes necessary for future research.

**Results:**

TDCS-related variation in serum metabolite levels was extremely small and statistically non-significant. Power calculations indicated that for the observed variation to be deemed significant, samples sizes of up to 11,000 subjects per group would be required, depending on the metabolite of interest.

**Conclusion:**

Our study found that five sessions of tDCS induced no major effects on peripheral metabolites among healthy men. These observations support the view of tDCS as a safe treatment that does not induce significant changes in the measured peripheral metabolites in healthy male subjects.

## Introduction

Transcranial direct current stimulation (tDCS) is a non-invasive method for modulating neuronal activity by introducing a small electric current into the brain via electrodes placed on the scalp. It has attracted increasing interest among both clinicians and researchers during the past decade. It has been speculated to be a promising candidate in the treatment of major depressive disorder (1), bipolar depression (2), dependence and craving (3), as well as neuropathic and idiopathic pain (3). The low cost, safety and simplicity of use make tDCS an attractive option for clinical applications, even though more research is needed to determine the optimal treatment protocols and patient characteristics.

At the cellular level, tDCS is considered to alter neuronal resting membrane potentials (4, 5). For example, anodal stimulation has been observed to lead to increased neuronal activity in the motor cortex, while cathodal stimulation of the same area has been considered to inhibit neuronal firing (5, 6). In addition to local cortical effects in neuronal excitability, recent research has demonstrated that tDCS also appears to exert changes in neuronal activity in deeper brain structures, such as the midbrain nuclei (7).

The brain acts as a control unit that regulates the functions of the entire organism, and is in turn modified by peripheral physiology. Nevertheless, only limited knowledge is available on possible peripheral changes induced by brain stimulation using tDCS. Previous research has indicated that tDCS induces changes in cerebral blood flow (8), brain neurotransmitter levels (9) and central and peripheral metabolic activity (10). Furthermore, anodal tDCS in the area corresponding to the left dorsolateral prefrontal cortex (DLPFC) has been postulated to lead to changes in autonomic nervous system activity, measured by high- and low-frequency heart rate variability and the secretion of cortisol under normal conditions (11), as well as in stressful situations (12, 13). Thus, current evidence indicates that tDCS may induce very specific physiological responses in both the central nervous system and the periphery, although our understanding of possible systemic metabolic adaptations is very limited.

Electrical fields created by tDCS exert force on polar and/or charged molecules, and many proteins and amino acids, which often serve as neurotransmitters of the brain, are charged. This could lead to alterations in the concentrations of polar molecules and trigger larger metabolic cascades. These consequences may explain some of the observed physiological effects of tDCS in both the central nervous system and the periphery.

With regards to the peripheral effects of tDCS, a previous study conducted by Binkofski et al. (10) indicated that anodal tDCS led to a transient drop in high-energy phosphorus compounds (adenosine triphosphate (ATP), phosphocreatinine) both under the electrode and in the contralateral hemisphere, suggesting that there had been a widespread increase in neuronal glutaminergic activity. By using a hyperinsulinemic–euglycemic clamp, they also observed a simultaneous increase in glucose uptake in the peripheral circulation. The extent of the increase clearly correlated with the levels of the previously mentioned energetic compounds in the brain tissue, suggestive of elevated glucose uptake across the BBB.

As many physiological processes in the central nervous system result in observable changes in metabolite profiles in the periphery, metabolomic analysis of possible tDCS effects could provide a valuable tool for investigating the safety profile and mechanisms of action of tDCS. As many circulating metabolites are biologically active compounds, alterations in the levels of such compounds could result in safety concerns in healthy or clinical populations. We investigated the acute effects of tDCS on peripheral metabolites in a sample of 79 healthy men. The working hypotheses were that the metabolic state of the participants would change as a result of the stimulation and that differences between the study groups could be reflected in altered metabolite profiles.

## Materials and Methods

### Study Population

This investigation formed part of the larger Optimizing Transcranial Electrical Stimulation for Clinical Applications (OptES) Study. The OptES Study was designed to generate novel information on the mechanisms of action of transcranial electrical stimulation, and to use this information to develop new, better clinical applications of transcranial electrical stimulation. The study protocol was approved by the Ethics Committee of the North Savo Hospital District (permit number 41/2015). All participants provided written informed consent after a full explanation of the study. The study conformed with the Declaration of Helsinki.

In order to decrease random variance in our findings, we chose to include only male participants, since our metabolomics platform also included some compounds known to be affected by sex hormones either centrally (14) or in the periphery (15, 16). A total of 80 male volunteers were recruited from the North Savo region of Finland. The participants received either tDCS or sham stimulation in a double-blind setting. The inclusion criteria were male gender (to avoid possible confounding metabolic effects of the menstrual cycle), aged between 18 and 40 years at the time of recruitment, right-handedness (i.e., belonging to the 1st to 10th right decile according to the Edinburgh Handedness Questionnaire (17)), and not having previously received tDCS. The exclusion criteria were metal implants inside the skull or eye, severe skin lesions in the electrode placement areas, a pacemaker, a history of epilepsy or previous seizures, a history of intracerebral bleeding during the previous six months, a self-reported history of substance dependence/abuse during the past six months and a history of any endocrinological condition [i.e., any physician-defined E00-E32 diagnosis according to the International Statistical Classification of Diseases and Related Health Problems version 10 (IDC-10) (18)]. We were unable to obtain the post-stimulation venous blood sample from one participant, and this individual was therefore excluded from all analyses. Thus, the final sample size was 40 in the tDCS group (mean age 28.3 years, mean BMI 26.0 kg/m^2^) and 39 in the sham group (mean age 27.7 years, mean BMI 25.4 kg/m^2^).

### Experimental Procedure

The participants were randomly assigned to either 1) the active stimulation or 2) the sham stimulation group in a 1:1 fashion, utilizing a computer-generated scheme. Basic sociodemographic information, including age, height and weight used to calculate the body mass index (BMI, kg/m^2^), was collected from questionnaires that the participants completed prior to the first stimulation. Three venous blood samples were collected via venipuncture by a trained nurse.

**Figure 1:**
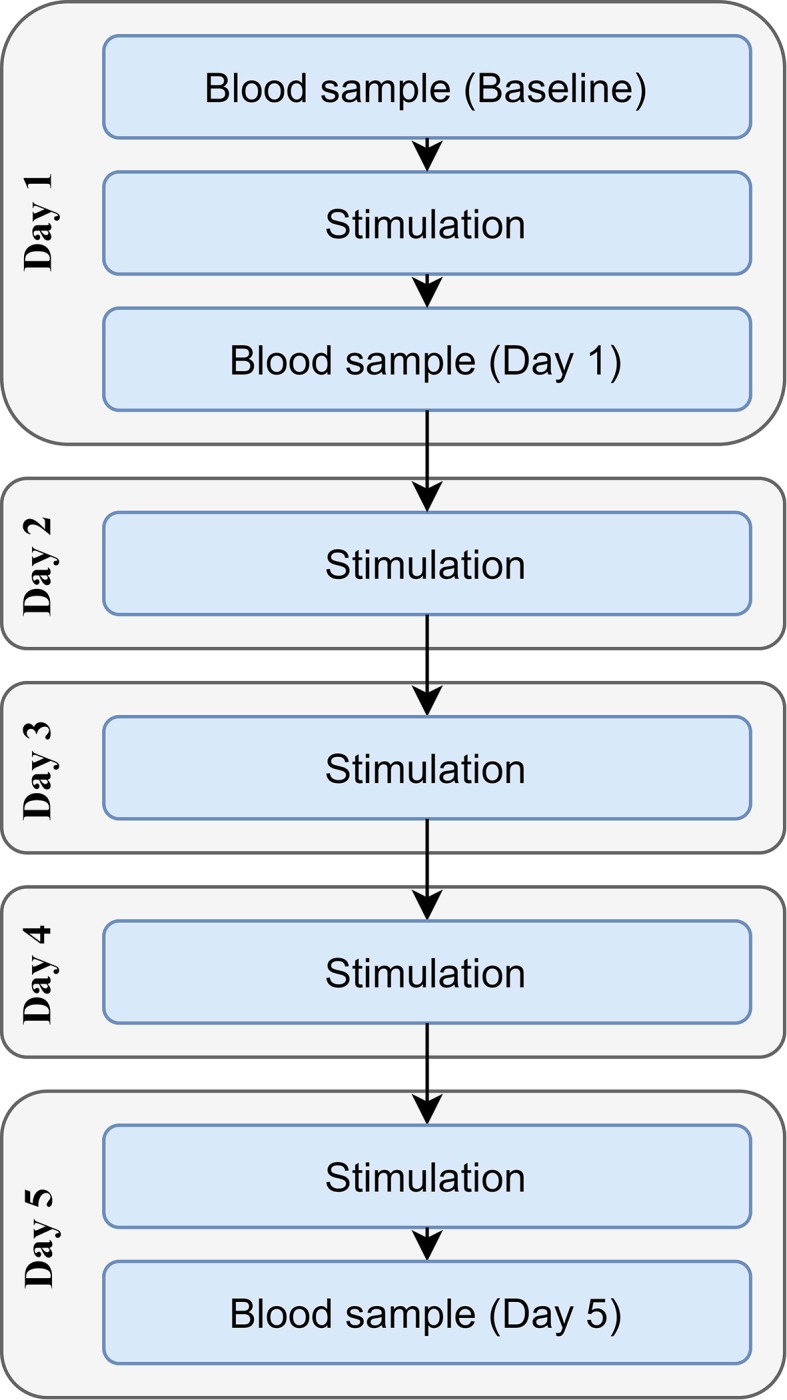
Timeline of the study.

Each participant received either tDCS or sham stimulation once per day for five consecutive days (**Figure 1**). Sociodemographic questionnaires were completed before the stimulation on day one. The baseline blood sample was collected immediately before the first stimulation session, with the second sample being drawn immediately after the first stimulation session (in order to investigate the acute effects of stimulation); the third sample was drawn immediately after the fifth stimulation session. A maximum of 5 min was allowed between stimulation and the collection of the blood samples.

Before the experiment, the participants were instructed to abstain from alcohol for 12 h and to have consumed no more than two doses during the preceding 24 h, to abstain from products containing caffeine for 3 h, and to abstain from smoking and heavy exercise for 1 h prior to the experiment. In addition, all experiments were conducted in the morning, and the participants were instructed to fast for 10 h to allow fasting blood samples to be obtained. This procedure enabled minimization of the confounding metabolic effects of the consumption of dietary products. Before stimulation, participant compliance with the instructions was checked, and those not conforming to the instructions were re-scheduled to another testing day.

Each participant received a 20-minute stimulation session with a current of 2 mA using a neuroConn DC-Stimulator (neuroConn GmbH, Ilmenau, Germany). The experimenter was unaware of the form of stimulation delivered (i.e., active stimulation vs. sham stimulation), and the participants were randomly divided between the study groups by a computer. The electrodes were made of conductive rubber placed inside sponge pads soaked with 12 ml of saline and held down with elastic straps. The electrode area was 25 cm^2^, resulting in a current density of 0.8 A/m^2^. The anode was placed at site F3 and the cathode at site F4 according to the international 10–20 electroencephalography system. In order to standardize the external stimuli, during the stimulation the participants were asked to refrain from talking and to watch a neutral landscape video with headphones on. The sham group received 15 seconds of ramping up and ramping down at the beginning, after which stimulation was discontinued.

After the stimulation, both the participant and the experimenter filled in a form in which they were asked to provide their estimate (percentage) of the likelihood of the participant belonging to the sham group.

### Blood Sample Analysis

Venous blood samples were collected into Vacuette 454078 4-ml serum gel tubes (Greiner Bio-One GmbH, Rainbach im Mühlkreis, Austria). They were left at room temperature for 30 min, followed by 10 min of centrifugation at 2,400×*g*, +20°C, to separate serum. The serum samples were frozen at −80°C until analysed. The metabolites were extracted from the serum samples using acetonitrile:formic acid (99:1 v/v) as a solvent (1:4, sample:solvent) and analysed using an ACQUITY UPLC-MS/MS system (Waters Corporation, Milford, MA, USA). A detailed protocol and instrument conditions have been published elsewhere (19).

### Statistical Methods

Preliminary inspections of the metabolomic data allowed the detection of a total of 2,124 missing observations (8.786% of the values in the dataset). Because an excessive number of missing values for some of the measured compounds could potentially introduce unanticipated biases, metabolites with ≥50% of missing values within either of the experimental groups (n = 9) were excluded from any further statistical testing (**Supplementary Table 1**, **Supplementary Figure 1**), and the remaining metabolites (n = 93) were included in the further analyses.

After appropriate transformation and baseline standardization, each metabolite was subsequently entered into a generalized estimating equations model to investigate differences between groups in their respective values at baseline and after tDCS or sham stimulations were applied. The p-value threshold for statistical significance was set at p ≤5.376e−04, as the complexity of the analyses developed required accounting for both the family-wise error rate (Bonferroni method) and the intrinsic correlated nature of the metabolite data. Further details of the models implemented can be found in the **Supplementary Methods** and **Supplementary Figure 2**

These main statistical analyses were subsequently supplemented with a set of power-related computations utilizing statistical simulations within a sophisticated computer cluster environment. Firstly, we evaluated the sensitivity obtained with the current sample in its ability to detect significant longitudinal differences between groups with ≥80% power (for 0.05 and 5.376e−04 type-I error rates). This would allow an estimation of the minimum level of detection with the current experimental set-up. Subsequently, for each metabolite, we evaluated the sample size that would be required to detect the variation in metabolites caused by tDCS in this study as statistically significant with ≥80% power (type-I error rates 0.05 and 5.376e−04). This was done in order to make an estimate of the sample size needed in future experimental settings aiming to detect similar changes in metabolites resulting from tDCS (assuming that the detected values in this experiment would apply to other healthy populations with similar characteristics). Further details of these computations are offered in the **Supplementary Methods**.

Lastly, the success of the blinding procedure was investigated by running a set of Mann–Whitney U-tests for both the participants and the experimenters on days one and five. They were asked how likely they thought it was, as a percentage, that they were part of the sham group, and the answers were used in the analysis.

## Results

**Figure 2:**
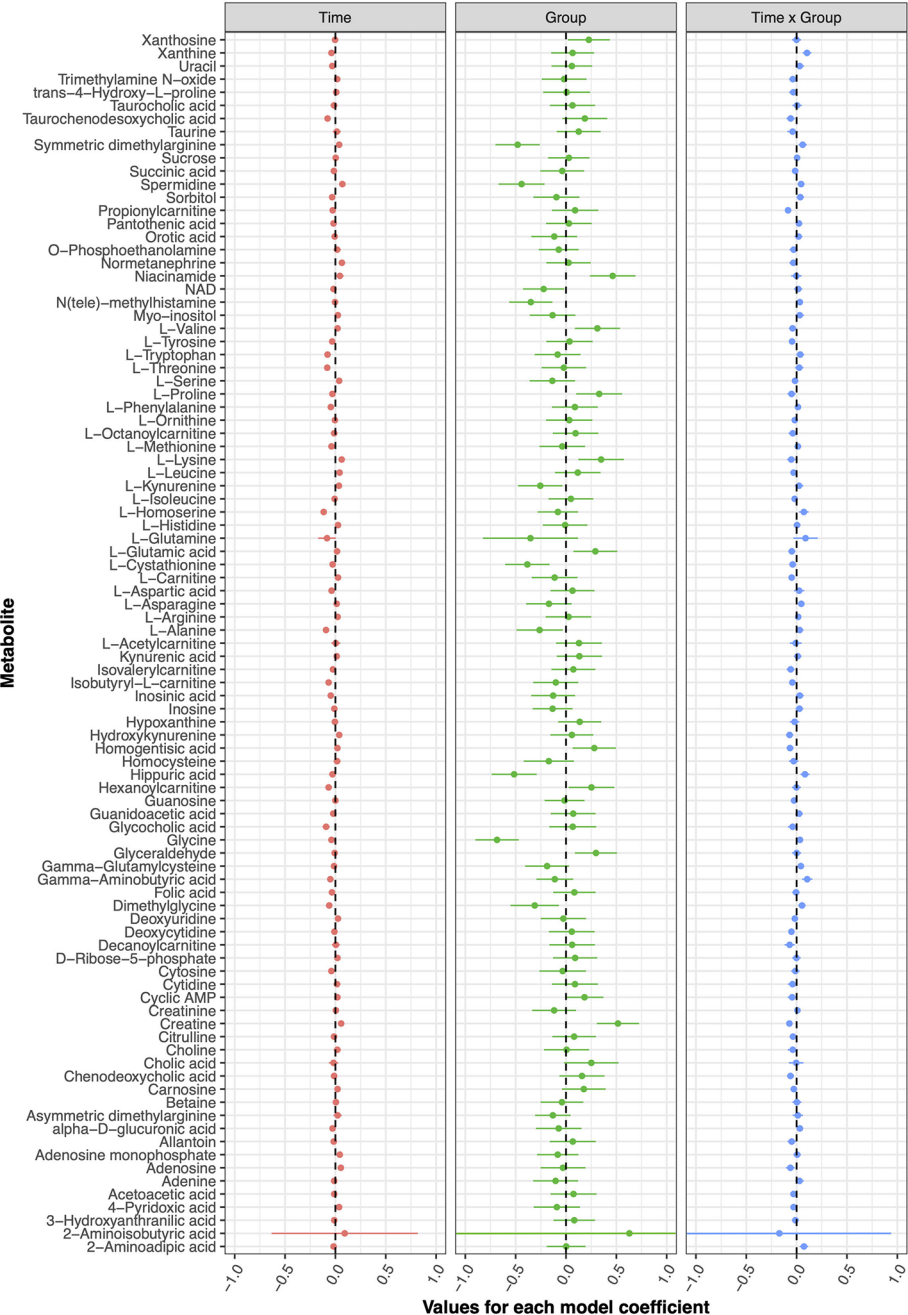
Coefficient values with 95% confidence intervals from each metabolite model analysis.

The current double-blind, randomized controlled trial failed to detect any metabolic changes due to tDCS after five treatment sessions; none of the models implemented for any of the investigated metabolites displayed statistically significant coefficients. **Figure 2** presents the model coefficients obtained for each metabolite, while the specific values provided by the statistical tests can be found in **Table 1**.

**Table 1:** Descriptive statistics from the pre-processed data, with main results from the models evaluated for each metabolite.

Metabolite Name	Baseline		Post session 1		Post session 5		Coefficient for “Time” **	Coefficient for “Group” ** ^	Coefficient for “Time × Group” **
	tDCS Group *	Sham Group *	tDCS Group *	Sham Group *	tDCS Group *	Sham Group *			
**2-Aminoadipic acid**	39; 0.012 (0.166)	39; −0.012 (0.156)	40; 0.092 (0.153)	39; 0.062 (0.177)	39; 0.228 (0.136)	39; −0.106 (0.151)	−0.018 (0.027); 0.434; 0.51	0.001 (0.193); 0; 0.994	0.073 (0.041); 3.113; 0.078
**2-Aminoisobutyric acid**	40; 0.127 (0.129)	39; −0.13 (0.185)	40; 0.137 (0.131)	39; −0.065 (0.18)	40; 0.283 (0.141)	39; 0.217 (0.156)	0.092 (0.726); 0.016; 0.899	0.629 (3.6); 0.031; 0.861	−0.172 (1.137); 0.023; 0.88
**3-Hydroxyanthranilic acid**	40; 0.055 (0.151)	39; −0.056 (0.169)	40; −0.051 (0.128)	39; −0.139 (0.168)	40; −0.082 (0.153)	39; −0.092 (0.143)	−0.011 (0.025); 0.206; 0.65	0.082 (0.206); 0.158; 0.691	−0.012 (0.036); 0.11; 0.74
**4-Pyridoxic acid**	40; −0.044 (0.121)	39; 0.045 (0.192)	40; -0.067 (0.118)	39; 0.034 (0.186)	40; −0.05 (0.16)	39; 0.214 (0.19)	0.037 (0.019); 3.726; 0.054	−0.091 (0.229); 0.158; 0.691	−0.03 (0.029); 1.032; 0.31
**Acetoacetic acid**	40; 0.005 (0.154)	39; −0.005 (0.167)	40; −0.044 (0.158)	39; −0.193 (0.174)	40; −0.223 (0.18)	39; −0.148 (0.163)	−0.012 (0.018); 0.413; 0.52	0.075 (0.228); 0.107; 0.744	−0.031 (0.028); 1.207; 0.272
**Adenine**	40; −0.021 (0.176)	39; 0.022 (0.142)	40; −0.174 (0.132)	39; 0.007 (0.195)	40; 0.04 (0.135)	39; −0.044 (0.134)	−0.012 (0.027); 0.205; 0.651	−0.103 (0.223); 0.215; 0.643	0.029 (0.042); 0.46; 0.498
**Adenosine**	40; −0.066 (0.164)	39; 0.067 (0.155)	40; 0.252 (0.163)	39; 0.225 (0.177)	40; 0.057 (0.186)	39; 0.378 (0.167)	0.054 (0.024); 4.931; 0.026	−0.03 (0.223); 0.018; 0.893	−0.061 (0.044); 1.963; 0.161
**Adenosine monophosphate**	40; 0.04 (0.142)	39; −0.041 (0.177)	40; 0.101 (0.138)	39; 0.349 (0.145)	40; 0.251 (0.123)	39; 0.211 (0.14)	0.044 (0.027); 2.721; 0.099	−0.082 (0.205); 0.161; 0.688	0.005 (0.038); 0.017; 0.895
**Allantoin**	40; 0.038 (0.173)	39; −0.039 (0.144)	40; −0.125 (0.174)	39; −0.103 (0.154)	40; −0.312 (0.177)	39; −0.177 (0.154)	−0.017 (0.025); 0.439; 0.507	0.067 (0.228); 0.086; 0.769	−0.049 (0.042); 1.348; 0.246
**alpha-D-glucuronic acid**	40; −0.016 (0.165)	39; 0.016 (0.155)	40; −0.176 (0.158)	39; −0.071 (0.172)	40; −0.054 (0.15)	39; −0.132 (0.142)	-0.028 (0.021); 1.87; 0.172	−0.073 (0.226); 0.103; 0.748	0.032 (0.034); 0.879; 0.348
**Asymmetric dimethylarginine**	40; −0.145 (0.16)	39; 0.149 (0.157)	40; 0.171 (0.135)	39; 0.062 (0.134)	40; 0.034 (0.132)	39; 0.236 (0.154)	0.022 (0.041); 0.282; 0.595	−0.13 (0.176); 0.547; 0.459	0.012 (0.051); 0.055; 0.815
**Betaine**	40; 0.008 (0.16)	39; −0.008 (0.16)	40; −0.083 (0.153)	39; 0.048 (0.133)	40; 0.01 (0.176)	39; 0.003 (0.119)	0.005 (0.027); 0.036; 0.85	−0.04 (0.212); 0.036; 0.849	0.003 (0.044); 0.004; 0.95
**Carnosine**	40; 0.114 (0.153)	39; −0.116 (0.165)	40; 0.05 (0.143)	39; 0.061 (0.134)	40; 0.064 (0.129)	39; −0.004 (0.115)	0.021 (0.025); 0.752; 0.386	0.177 (0.217); 0.662; 0.416	−0.028 (0.033); 0.711; 0.399
**Chenodeoxycholic acid**	40; 0.046 (0.143)	39; −0.047 (0.176)	40; 0.026 (0.138)	39; −0.14 (0.167)	40; −0.286 (0.123)	39; −0.133 (0.186)	−0.013 (0.023); 0.321; 0.571	0.159 (0.224); 0.501; 0.479	−0.061 (0.034); 3.145; 0.076
**Cholic acid**	30; 0.08 (0.186)	28; −0.086 (0.188)	26; −0.159 (0.209)	25; −0.175 (0.161)	27; 0.002 (0.194)	23; −0.387 (0.202)	−0.018 (0.047); 0.151; 0.697	0.252 (0.27); 0.867; 0.352	−0.004 (0.072); 0.003; 0.955
**Choline**	40; 0.03 (0.148)	39; −0.031 (0.171)	40; −0.024 (0.175)	39; −0.044 (0.157)	40; −0.121 (0.158)	39; 0.05 (0.197)	0.019 (0.036); 0.287; 0.592	0.006 (0.223); 0.001; 0.979	−0.038 (0.05); 0.578; 0.447
**Citrulline**	40; 0.035 (0.166)	39; −0.036 (0.154)	40; −0.127 (0.152)	39; −0.163 (0.151)	40; −0.229 (0.182)	39; −0.164 (0.18)	−0.014 (0.026); 0.284; 0.594	0.081 (0.217); 0.138; 0.71	−0.036 (0.034); 1.085; 0.298
**Creatine**	40; 0.302 (0.151)	39; −0.309 (0.154)	40; 0.308 (0.146)	39; −0.074 (0.133)	40; 0.229 (0.141)	39; −0.051 (0.137)	0.057 (0.019); 8.719; 0.003	0.516 (0.21); 6.014; 0.014	−0.072 (0.028); 6.578; 0.01
**Creatinine**	40; −0.087 (0.154)	39; 0.089 (0.165)	40; −0.098 (0.149)	39; −0.024 (0.157)	40; −0.026 (0.164)	39; 0.076 (0.176)	0.005 (0.015); 0.098; 0.754	−0.118 (0.218); 0.293; 0.588	0.008 (0.022); 0.129; 0.72
**Cyclic AMP**	40; 0.129 (0.174)	39; −0.133 (0.141)	40; 0.086 (0.141)	39; −0.054 (0.14)	40; 0.043 (0.135)	39; 0.021 (0.141)	0.02 (0.023); 0.77; 0.38	0.184 (0.188); 0.956; 0.328	−0.044 (0.045); 0.967; 0.326
**Cytidine**	40; 0.038 (0.14)	39; −0.039 (0.179)	40; −0.013 (0.133)	39; −0.054 (0.177)	40; −0.045 (0.167)	39; 0.066 (0.195)	0.016 (0.035); 0.207; 0.649	0.089 (0.228); 0.153; 0.696	−0.04 (0.046); 0.762; 0.383
**Cytosine**	40; −0.013 (0.18)	39; 0.013 (0.137)	40; −0.079 (0.16)	39; 0.015 (0.139)	40; −0.304 (0.16)	39; −0.232 (0.14)	−0.04 (0.026); 2.348; 0.125	−0.033 (0.232); 0.021; 0.886	−0.012 (0.042); 0.084; 0.772
**D-Ribose-5-phosphate**	40; 0.053 (0.162)	39; −0.054 (0.157)	40; 0.038 (0.147)	39; 0.02 (0.144)	40; 0.129 (0.128)	39; −0.023 (0.14)	0.02 (0.027); 0.555; 0.456	0.09 (0.22); 0.17; 0.68	−0.002 (0.042); 0.002; 0.969
**Decanoylcarnitine**	40; 0.04 (0.156)	39; −0.041 (0.165)	40; −0.105 (0.18)	39; −0.401 (0.18)	40; −0.36 (0.173)	39; −0.287 (0.112)	0.005 (0.039); 0.015; 0.902	0.061 (0.226); 0.072; 0.788	−0.069 (0.051); 1.878; 0.171
**Deoxycytidine**	40; 0.018 (0.128)	39; −0.018 (0.188)	40; −0.036 (0.123)	39; −0.127 (0.184)	40; −0.267 (0.136)	39; −0.044 (0.177)	−0.01 (0.014); 0.528; 0.468	0.057 (0.227); 0.064; 0.8	−0.051 (0.025); 4.151; 0.042
**Deoxyuridine**	40; −0.02 (0.151)	39; 0.02 (0.169)	40; 0.058 (0.153)	39; −0.025 (0.17)	40; 0.031 (0.139)	39; 0.121 (0.189)	0.026 (0.02); 1.6; 0.206	−0.026 (0.225); 0.013; 0.908	−0.018 (0.029); 0.372; 0.542
**Dimethylglycine**	40; −0.106 (0.145)	39; 0.109 (0.173)	40; −0.129 (0.145)	39; -0.007 (0.162)	40; −0.089 (0.13)	39; 0.112 (0.169)	−0.061 (0.031); 3.848; 0.05	−0.311 (0.24); 1.673; 0.196	0.053 (0.037); 2.086; 0.149
**Folic acid**	40; 0.063 (0.175)	39; −0.064 (0.142)	40; 0.181 (0.16)	39; 0.016 (0.134)	40; −0.053 (0.184)	39; −0.169 (0.148)	−0.034 (0.026); 1.706; 0.191	0.083 (0.21); 0.156; 0.693	−0.006 (0.037); 0.025; 0.875
**Gamma-Aminobutyric acid**	40; −0.116 (0.163)	39; 0.119 (0.155)	40; 0.004 (0.155)	39; −0.102 (0.149)	40; 0.184 (0.148)	39; −0.18 (0.129)	−0.05 (0.032); 2.479; 0.115	−0.112 (0.183); 0.372; 0.542	0.105 (0.051); 4.207; 0.04
**Gamma-Glutamylcysteine**	38; −0.05 (0.169)	34; 0.055 (0.165)	37; −0.205 (0.133)	38; 0.035 (0.175)	38; 0.014 (0.159)	34; −0.005 (0.145)	−0.014 (0.019); 0.505; 0.477	−0.188 (0.218); 0.743; 0.389	0.042 (0.029); 2.127; 0.145
**Glyceraldehyde**	40; 0.167 (0.143)	39; −0.171 (0.172)	40; −0.089 (0.135)	39; −0.271 (0.163)	40; 0.089 (0.139)	39; −0.243 (0.154)	−0.007 (0.029); 0.064; 0.8	0.298 (0.209); 2.026; 0.155	0.001 (0.045); 0; 0.991
**Glycine**	40; −0.296 (0.169)	39; 0.304 (0.133)	40; −0.319 (0.177)	39; 0.36 (0.143)	40; −0.304 (0.183)	39; 0.094 (0.125)	−0.039 (0.018); 4.72; 0.03	−0.685 (0.216); 10.095; 0.001	0.033 (0.029); 1.291; 0.256
**Glycocholic acid**	40; 0.011 (0.165)	39; −0.012 (0.155)	40; −0.31 (0.172)	39; −0.465 (0.164)	40; −0.61 (0.163)	39; −0.467 (0.153)	−0.092 (0.036); 6.521; 0.011	0.067 (0.233); 0.082; 0.775	−0.039 (0.048); 0.681; 0.409
**Guanidoacetic acid**	40; 0.028 (0.149)	39; −0.029 (0.171)	40; −0.159 (0.144)	39; −0.209 (0.169)	40; 0.005 (0.2)	39; −0.216 (0.187)	−0.021 (0.023); 0.838; 0.36	0.071 (0.223); 0.102; 0.749	0.025 (0.037); 0.439; 0.508
**Guanosine**	40; −0.068 (0.159)	39; 0.07 (0.16)	40; 0.179 (0.133)	39; 0.023 (0.161)	40; −0.064 (0.137)	39; 0.06 (0.161)	0 (0.022); 0; 0.994	−0.014 (0.198); 0.005; 0.943	−0.026 (0.031); 0.681; 0.409
**Hexanoylcarnitine**	40; 0.133 (0.162)	39; −0.136 (0.155)	40; −0.165 (0.161)	39; −0.496 (0.165)	40; −0.251 (0.177)	39; −0.45 (0.127)	−0.067 (0.031); 4.866; 0.027	0.253 (0.227); 1.235; 0.266	−0.002 (0.044); 0.002; 0.961
**Hippuric acid**	40; −0.254 (0.145)	39; 0.261 (0.164)	40; −0.451 (0.135)	39; 0.039 (0.159)	40; 0.003 (0.192)	39; 0.116 (0.167)	−0.029 (0.034); 0.718; 0.397	−0.515 (0.224); 5.288; 0.021	0.083 (0.045); 3.437; 0.064
**Homocysteine**	40; -0.08 (0.15)	39; 0.082 (0.169)	40; −0.094 (0.156)	39; 0.088 (0.168)	40; −0.118 (0.146)	39; 0.125 (0.158)	0.017 (0.035); 0.235; 0.628	−0.171 (0.25); 0.468; 0.494	−0.029 (0.047); 0.387; 0.534
**Homogentisic acid**	40; 0.144 (0.165)	39; −0.147 (0.151)	40; −0.06 (0.135)	38; −0.261 (0.183)	40; −0.131 (0.131)	39; −0.102 (0.137)	0.02 (0.022); 0.865; 0.352	0.281 (0.215); 1.705; 0.192	−0.066 (0.034); 3.668; 0.055
**Hydroxykynurenine**	40; 0.053 (0.158)	39; −0.055 (0.161)	40; −0.004 (0.128)	39; −0.043 (0.171)	40; −0.092 (0.154)	39; 0.143 (0.156)	0.038 (0.025); 2.291; 0.13	0.058 (0.213); 0.074; 0.785	−0.069 (0.037); 3.59; 0.058
**Hypoxanthine**	40; 0.135 (0.155)	39; −0.138 (0.163)	40; −0.129 (0.117)	39; −0.02 (0.141)	40; 0.041 (0.131)	39; −0.181 (0.119)	−0.005 (0.033); 0.022; 0.883	0.136 (0.214); 0.404; 0.525	−0.02 (0.046); 0.19; 0.663
**Inosine**	40; −0.109 (0.159)	39; 0.112 (0.159)	40; 0.154 (0.12)	39; 0.083 (0.155)	40; 0.062 (0.148)	39; 0.055 (0.16)	−0.011 (0.028); 0.155; 0.694	−0.133 (0.198); 0.451; 0.502	0.028 (0.039); 0.526; 0.468
**Inosinic acid**	40; 0.008 (0.17)	39; −0.008 (0.149)	40; −0.085 (0.171)	39; 0.136 (0.161)	40; −0.113 (0.174)	39; −0.159 (0.159)	−0.046 (0.022); 4.373; 0.037	−0.128 (0.218); 0.344; 0.558	0.032 (0.04); 0.641; 0.423
**Isobutyryl-L-carnitine**	40; −0.09 (0.145)	39; 0.092 (0.174)	40; −0.285 (0.134)	39; −0.131 (0.165)	40; −0.555 (0.139)	39; −0.192 (0.175)	−0.067 (0.022); 9.072; 0.003	−0.102 (0.225); 0.206; 0.65	−0.042 (0.031); 1.906; 0.167
**Isovalerylcarnitine**	40; 0.035 (0.134)	39; −0.035 (0.183)	40; −0.001 (0.128)	39; −0.214 (0.176)	40; −0.385 (0.145)	39; −0.147 (0.154)	−0.025 (0.03); 0.698; 0.403	0.073 (0.219); 0.112; 0.738	−0.059 (0.041); 2.097; 0.148
**Kynurenic acid**	40; 0.057 (0.137)	39; −0.059 (0.181)	40; −0.065 (0.133)	39; −0.23 (0.167)	40; 0.118 (0.155)	39; −0.066 (0.175)	0.013 (0.027); 0.236; 0.627	0.133 (0.225); 0.351; 0.554	0.011 (0.038); 0.079; 0.779
**L-Acetylcarnitine**	40; 0.049 (0.154)	39; −0.05 (0.166)	40; −0.027 (0.171)	39; −0.111 (0.168)	40; 0.044 (0.2)	39; −0.009 (0.16)	0.004 (0.044); 0.007; 0.931	0.129 (0.227); 0.322; 0.57	−0.009 (0.058); 0.023; 0.88
**L-Alanine**	40; −0.119 (0.166)	39; 0.122 (0.151)	40; −0.152 (0.184)	39; 0.02 (0.142)	40; −0.485 (0.168)	39; −0.373 (0.149)	−0.093 (0.028); 10.741; 0.001	−0.262 (0.23); 1.298; 0.255	0.029 (0.039); 0.569; 0.451
**L-Arginine**	40; 0.023 (0.153)	39; −0.024 (0.167)	40; 0.054 (0.149)	39; −0.008 (0.151)	40; 0.225 (0.148)	39; 0.097 (0.172)	0.022 (0.019); 1.408; 0.235	0.025 (0.226); 0.012; 0.912	0.016 (0.03); 0.277; 0.599
**L-Asparagine**	40; −0.08 (0.166)	39; 0.082 (0.152)	40; −0.129 (0.181)	39; 0.023 (0.155)	40; 0.13 (0.167)	39; 0.092 (0.161)	0.013 (0.025); 0.261; 0.61	−0.17 (0.226); 0.564; 0.453	0.046 (0.032); 2.072; 0.15
**L-Aspartic acid**	40; 0.107 (0.165)	39; −0.109 (0.153)	40; −0.375 (0.143)	39; −0.205 (0.163)	40; −0.091 (0.175)	39; −0.333 (0.157)	−0.038 (0.031); 1.457; 0.227	0.065 (0.219); 0.087; 0.767	0.025 (0.051); 0.241; 0.624
**L-Carnitine**	40; −0.027 (0.16)	39; 0.028 (0.16)	40; −0.065 (0.163)	39; 0.059 (0.154)	40; −0.128 (0.17)	39; 0.159 (0.154)	0.026 (0.014); 3.701; 0.054	−0.113 (0.227); 0.248; 0.618	−0.049 (0.02); 5.801; 0.016
**L-Cystathionine**	40; -0.184 (0.13)	39; 0.189 (0.181)	40; −0.378 (0.122)	39; 0.058 (0.187)	40; −0.457 (0.117)	39; 0.09 (0.175)	−0.027 (0.023); 1.368; 0.242	−0.384 (0.22); 3.037; 0.081	−0.039 (0.035); 1.229; 0.268
**L-Glutamic acid**	40; 0.159 (0.171)	39; −0.163 (0.143)	40; 0.062 (0.136)	39; −0.033 (0.152)	40; 0.053 (0.126)	39; −0.198 (0.131)	0.017 (0.022); 0.564; 0.453	0.292 (0.218); 1.788; 0.181	−0.047 (0.036); 1.776; 0.183
**L-Glutamine**	40; −0.089 (0.15)	39; 0.091 (0.169)	40; −0.04 (0.148)	39; 0.136 (0.17)	40; 0.064 (0.136)	39; 0.095 (0.153)	−0.083 (0.088); 0.896; 0.344	−0.353 (0.473); 0.558; 0.455	0.088 (0.121); 0.53; 0.467
**L-Histidine**	40; 0.006 (0.161)	39; −0.006 (0.159)	40; 0.013 (0.144)	39; −0.036 (0.16)	40; 0.155 (0.164)	39; 0.104 (0.176)	0.026 (0.025); 1.094; 0.296	−0.009 (0.221); 0.002; 0.968	0.004 (0.035); 0.015; 0.903
**L-Homoserine**	40; −0.017 (0.167)	39; 0.017 (0.152)	40; −0.243 (0.156)	39; −0.11 (0.145)	40; −0.319 (0.155)	39; −0.604 (0.148)	−0.116 (0.028); 17.574; 0	−0.081 (0.201); 0.161; 0.688	0.072 (0.047); 2.341; 0.126
**L-Isoleucine**	40; 0.043 (0.161)	39; −0.044 (0.158)	40; −0.162 (0.133)	39; −0.154 (0.171)	40; −0.13 (0.144)	39; −0.122 (0.155)	−0.008 (0.014); 0.29; 0.59	0.048 (0.223); 0.047; 0.828	−0.02 (0.027); 0.516; 0.473
**L-Kynurenine**	40; −0.082 (0.148)	39; 0.084 (0.171)	40; −0.128 (0.138)	39; 0.101 (0.163)	40; 0.159 (0.159)	39; 0.252 (0.191)	0.035 (0.032); 1.198; 0.274	−0.255 (0.221); 1.33; 0.249	0.024 (0.043); 0.307; 0.58
**L-Leucine**	40; 0.051 (0.151)	39; −0.052 (0.169)	40; −0.102 (0.167)	39; −0.24 (0.161)	40; 0.05 (0.169)	39; 0.049 (0.188)	0.042 (0.016); 6.525; 0.011	0.117 (0.226); 0.266; 0.606	−0.03 (0.028); 1.136; 0.286
**L-Lysine**	40; 0.184 (0.152)	39; −0.188 (0.163)	40; 0.266 (0.162)	39; −0.151 (0.159)	40; 0.236 (0.159)	39; 0.061 (0.202)	0.062 (0.029); 4.611; 0.032	0.349 (0.226); 2.375; 0.123	−0.053 (0.042); 1.572; 0.21
**L-Methionine**	40; 0.015 (0.154)	39; −0.016 (0.166)	40; −0.182 (0.151)	39; −0.086 (0.169)	40; −0.103 (0.16)	39; −0.224 (0.148)	−0.039 (0.022); 3.154; 0.076	−0.037 (0.227); 0.027; 0.87	0.012 (0.035); 0.118; 0.731
**L-Octanoylcarnitine**	40; 0.049 (0.162)	39; −0.05 (0.158)	40; −0.032 (0.187)	39; −0.292 (0.163)	40; −0.236 (0.179)	39; −0.237 (0.115)	−0.012 (0.033); 0.131; 0.717	0.095 (0.226); 0.176; 0.675	−0.037 (0.043); 0.768; 0.381
**L-Ornithine**	40; 0.051 (0.161)	39; −0.052 (0.158)	40; −0.152 (0.167)	39; −0.094 (0.168)	40; −0.161 (0.169)	39; −0.086 (0.155)	−0.005 (0.019); 0.059; 0.809	0.032 (0.23); 0.019; 0.889	−0.019 (0.031); 0.373; 0.541
**L-Phenylalanine**	40; 0.061 (0.167)	39; −0.062 (0.151)	40; −0.129 (0.145)	39; −0.225 (0.145)	40; 0.011 (0.153)	39; −0.201 (0.14)	−0.046 (0.014); 10.562; 0.001	0.088 (0.228); 0.15; 0.698	0.013 (0.03); 0.194; 0.66
**L-Proline**	40; 0.162 (0.171)	39; −0.166 (0.143)	40; 0.06 (0.169)	39; −0.163 (0.132)	40; −0.212 (0.186)	39; −0.355 (0.153)	−0.03 (0.031); 0.936; 0.333	0.33 (0.228); 2.091; 0.148	−0.05 (0.041); 1.504; 0.22
**L-Serine**	40; −0.006 (0.129)	39; 0.006 (0.188)	40; −0.056 (0.127)	39; 0.168 (0.194)	40; 0.093 (0.145)	39; 0.233 (0.18)	0.037 (0.026); 2.04; 0.153	−0.136 (0.226); 0.363; 0.547	−0.016 (0.033); 0.23; 0.632
**L-Threonine**	40; 0.004 (0.149)	39; −0.004 (0.171)	40; −0.098 (0.143)	39; −0.047 (0.169)	40; −0.241 (0.189)	39; −0.443 (0.137)	−0.081 (0.026); 10.062; 0.002	−0.022 (0.221); 0.01; 0.922	0.026 (0.042); 0.375; 0.54
**L-Tryptophan**	40; −0.009 (0.138)	39; 0.009 (0.18)	40; −0.272 (0.145)	39; −0.262 (0.174)	40; −0.179 (0.154)	39; −0.298 (0.151)	−0.078 (0.029); 7.173; 0.007	−0.083 (0.228); 0.134; 0.714	0.036 (0.038); 0.871; 0.351
**L-Tyrosine**	40; 0.035 (0.153)	39; −0.036 (0.167)	40; −0.225 (0.16)	39; −0.226 (0.162)	40; −0.323 (0.155)	39; −0.216 (0.187)	−0.032 (0.018); 3.203; 0.073	0.035 (0.23); 0.023; 0.879	−0.045 (0.028); 2.544; 0.111
**L-Valine**	40; 0.148 (0.138)	39; −0.152 (0.177)	40; −0.008 (0.145)	39; −0.282 (0.164)	40; 0.025 (0.168)	39; −0.096 (0.169)	0.022 (0.025); 0.745; 0.388	0.312 (0.225); 1.926; 0.165	−0.04 (0.037); 1.185; 0.276
**Myo-inositol**	40; −0.009 (0.156)	39; 0.01 (0.164)	40; −0.171 (0.142)	39; 0.024 (0.196)	40; 0.181 (0.16)	39; 0.09 (0.163)	0.024 (0.027); 0.833; 0.361	−0.133 (0.227); 0.345; 0.557	0.031 (0.043); 0.517; 0.472
**N(tele)-methylhistamine**	40; −0.108 (0.158)	39; 0.111 (0.161)	40; −0.047 (0.148)	39; 0.175 (0.15)	40; 0.016 (0.152)	39; 0.094 (0.162)	−0.004 (0.013); 0.072; 0.788	−0.349 (0.214); 2.66; 0.103	0.032 (0.023); 1.863; 0.172
**NAD**	40; −0.08 (0.168)	39; 0.082 (0.15)	40; −0.056 (0.135)	39; 0.135 (0.177)	40; −0.08 (0.133)	39; 0.022 (0.156)	−0.019 (0.025); 0.593; 0.441	−0.222 (0.205); 1.178; 0.278	0.015 (0.042); 0.134; 0.714
**Niacinamide**	40; 0.23 (0.143)	39; −0.236 (0.168)	40; 0.929 (0.189)	39; 0.394 (0.147)	40; 0.377 (0.162)	39; −0.08 (0.175)	0.045 (0.037); 1.425; 0.233	0.463 (0.226); 4.194; 0.041	−0.003 (0.052); 0.003; 0.958
**Normetanephrine**	40; 0.053 (0.176)	39; −0.054 (0.142)	40; 0.287 (0.145)	39; 0.376 (0.177)	40; 0.22 (0.144)	39; 0.301 (0.148)	0.065 (0.023); 7.89; 0.005	0.026 (0.221); 0.013; 0.908	−0.032 (0.039); 0.672; 0.412
**O-Phosphoethanolamine**	40; 0.002 (0.136)	39; −0.002 (0.182)	40; −0.053 (0.138)	39; 0.079 (0.122)	40; −0.049 (0.143)	39; 0.075 (0.15)	0.018 (0.036); 0.242; 0.623	−0.072 (0.198); 0.133; 0.716	−0.029 (0.041); 0.508; 0.476
**Orotic acid**	40; 0.087 (0.155)	39; −0.089 (0.164)	40; −0.139 (0.187)	39; 0.256 (0.219)	40; 0.026 (0.158)	39; 0.045 (0.161)	−0.007 (0.029); 0.058; 0.809	−0.117 (0.228); 0.264; 0.607	0.019 (0.039); 0.237; 0.626
**Pantothenic acid**	40; 0.058 (0.153)	39; −0.059 (0.166)	40; −0.074 (0.151)	39; −0.054 (0.191)	40; 0.011 (0.157)	39; −0.123 (0.193)	−0.018 (0.023); 0.652; 0.419	0.029 (0.227); 0.016; 0.899	0.023 (0.033); 0.499; 0.48
**Propionylcarnitine**	40; 0.051 (0.138)	39; −0.052 (0.18)	40; −0.168 (0.141)	39; −0.235 (0.174)	40; −0.404 (0.142)	39; −0.103 (0.18)	−0.027 (0.017); 2.461; 0.117	0.09 (0.231); 0.153; 0.696	−0.085 (0.026); 10.381; 0.001
**Sorbitol**	40; −0.02 (0.184)	39; 0.02 (0.13)	40; −0.199 (0.187)	39; −0.012 (0.133)	40; 0.028 (0.188)	39; −0.174 (0.142)	−0.032 (0.017); 3.476; 0.062	−0.095 (0.228); 0.173; 0.677	0.036 (0.037); 0.962; 0.327
**Spermidine**	40; −0.156 (0.173)	39; 0.16 (0.14)	40; −0.033 (0.171)	39; 0.47 (0.181)	40; 0.378 (0.191)	39; 0.562 (0.162)	0.069 (0.024); 8.464; 0.004	−0.441 (0.229); 3.722; 0.054	0.045 (0.035); 1.636; 0.201
**Succinic acid**	40; −0.057 (0.157)	39; 0.058 (0.163)	40; −0.282 (0.158)	39; −0.253 (0.152)	40; −0.27 (0.141)	39; −0.121 (0.171)	−0.015 (0.022); 0.47; 0.493	−0.038 (0.218); 0.03; 0.863	−0.014 (0.032); 0.198; 0.656
**Sucrose**	40; 0.054 (0.156)	39; −0.056 (0.164)	40; −0.049 (0.16)	39; 0.088 (0.134)	40; 0.068 (0.147)	39; −0.039 (0.144)	0.003 (0.02); 0.022; 0.882	0.029 (0.206); 0.02; 0.889	0.004 (0.03); 0.021; 0.885
**Symmetric dimethylarginine**	40; −0.224 (0.161)	39; 0.23 (0.15)	40; −0.169 (0.154)	39; 0.113 (0.148)	40; 0.201 (0.154)	39; 0.378 (0.134)	0.037 (0.026); 2.134; 0.144	−0.481 (0.22); 4.752; 0.029	0.06 (0.04); 2.222; 0.136
**Taurine**	40; 0.102 (0.158)	39; −0.104 (0.16)	40; −0.285 (0.138)	39; −0.114 (0.155)	40; −0.057 (0.161)	39; −0.079 (0.159)	0.014 (0.04); 0.129; 0.72	0.126 (0.218); 0.334; 0.563	−0.039 (0.052); 0.575; 0.448
**Taurochenodesoxycholic acid**	40; 0.057 (0.171)	39; −0.059 (0.147)	40; −0.222 (0.158)	39; −0.401 (0.129)	40; −0.487 (0.131)	39; −0.332 (0.145)	−0.078 (0.03); 6.61; 0.01	0.187 (0.223); 0.705; 0.401	−0.059 (0.042); 1.97; 0.16
**Taurocholic acid**	40; 0.02 (0.138)	39; −0.021 (0.18)	40; −0.064 (0.138)	39; −0.152 (0.17)	40; 0.011 (0.16)	39; −0.047 (0.186)	−0.014 (0.035); 0.155; 0.694	0.064 (0.224); 0.083; 0.773	0.005 (0.047); 0.01; 0.919
**trans-4-Hydroxy-L-proline**	40; 0.012 (0.165)	39; −0.012 (0.154)	40; 0.01 (0.163)	39; 0.007 (0.152)	40; −0.301 (0.159)	39; −0.166 (0.165)	0.008 (0.033); 0.062; 0.803	0.006 (0.233); 0.001; 0.979	−0.032 (0.043); 0.555; 0.456
**Trimethylamine N-oxide**	40; −0.046 (0.144)	39; 0.047 (0.175)	40; −0.058 (0.139)	39; 0.099 (0.181)	40; −0.131 (0.147)	39; 0.043 (0.178)	0.018 (0.03); 0.347; 0.556	−0.019 (0.223); 0.007; 0.931	−0.037 (0.038); 0.934; 0.334
**Uracil**	40; 0.038 (0.177)	39; −0.039 (0.141)	40; −0.165 (0.176)	39; −0.158 (0.154)	40; −0.011 (0.134)	39; −0.235 (0.155)	−0.031 (0.031); 0.986; 0.321	0.058 (0.202); 0.083; 0.773	0.03 (0.044); 0.482; 0.488
**Xanthine**	40; 0.107 (0.155)	39; −0.11 (0.163)	40; −0.092 (0.145)	39; −0.092 (0.169)	40; 0.278 (0.158)	39; −0.296 (0.135)	−0.039 (0.03); 1.726; 0.189	0.065 (0.212); 0.095; 0.758	0.103 (0.042); 5.941; 0.015
**Xanthosine**	40; 0.138 (0.153)	39; −0.141 (0.164)	40; 0.086 (0.166)	39; 0.023 (0.144)	40; 0.064 (0.177)	39; −0.155 (0.149)	−0.003 (0.024); 0.018; 0.893	0.227 (0.209); 1.183; 0.277	−0.001 (0.044); 0; 0.989

*values represent: “N; Mean (SE)”; **values represent: “Estimate (SE); Wald statistic; P-value”; **^**“Group” coefficient specified in the model as variation in the tDCS group respect to the Sham.

The computations related to statistical power made it possible to draw relevant conclusions concerning the usability of the current data. Firstly, the conducted analyses suggested that our study sample conferred enough power (80% level) to detect relatively small differences between groups due to tDCS (an absolute value for the “Time x Group” model coefficient of ≥0.1493 would be detectable with a type-I error rate of 5.376 · 10^−4^). However, the majority of the tDCS effects observed here were substantially smaller than that threshold, and only a single metabolite, 2-aminoisobutyric acid, surpassed it. However, this metabolite was not ultimately considered as significant, because the model estimates displayed high uncertainties (for instance, see the extremely large standard error for the corresponding coefficients in **Table 1**).

**Figure 3:**
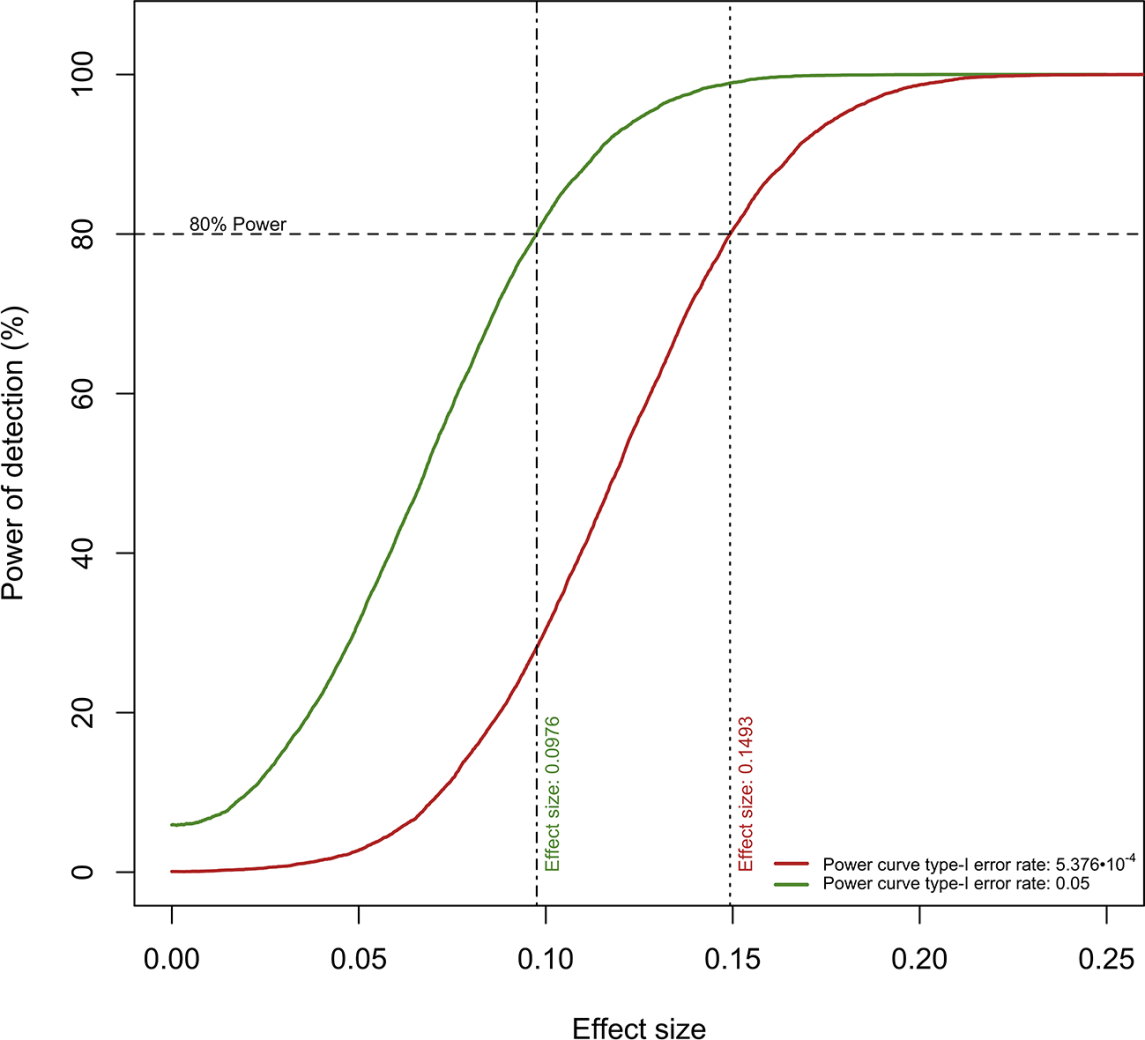
Power curves for the detection of “Time × Group” coefficient values. The current sample size per experimental group confers ≥80% power to detect “*Time Group*” coefficients of ≥0.0976 (type-I error rate: 0.05) and ≥0.1493 (type-I error rate: 5.376 · 10^−4^).

**Figure 4:**
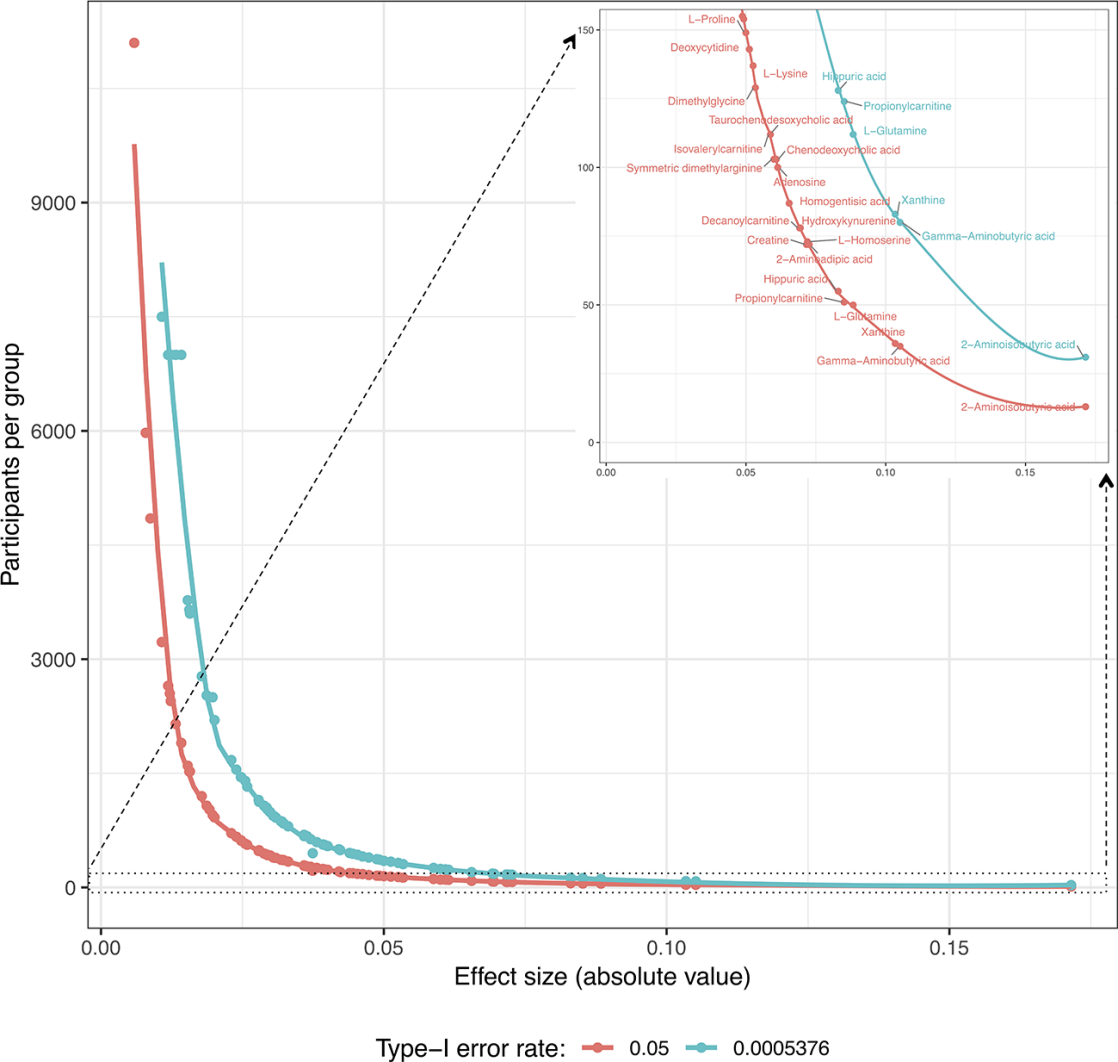
Estimation of the number of participants per group required for the significant detection of “*Time × Group*” coefficients (type-I error rates: 0.05 and 5.576 · 10^−4^; minimum detection power: 80%) for each metabolite according to the coefficient values obtained in these study models. In the upper right corner, there is a resized section of the overall curves clarifying the metabolites for which the estimated number of participants per group is ≤150.

Secondly, future replication studies aiming to detect significant group differences due to tDCS will need to increase their sample size considerably, as the treatment effects detected in our data were extremely small. For example, increasing the sample size to n = 150 participants per group (i.e., n = 300 in total) would have made it possible to identify six metabolites as displaying statistically significant differences between the study groups: 2-aminoisobutyric acid and propionylcarnitine being upregulated and hippuric acid, L-glutamine, xanthine and gamma-aminobutyric acid being downregulated in the tDCS group. **Figures 3** and **4** provide more details on this sample size estimation. Detailed results from the calculations are available in **Supplementary Table 2**.

Finally, it should be acknowledged here that the blinding protocol was not totally successful, as a larger number of participants and experimenters on day 1 compared to day 5 successfully guessed that they belonged to the sham group (p = 0.041 for participants, 0.012 for experimenters).

## Discussion

This investigation revealed that five daily sessions of tDCS applied to healthy male adults exerted no significant impact on serum metabolite levels when compared to sham stimulation. Although our study was adequately powered to detect relatively small effect sizes (more specifically, ≥0.1493), tDCS produced either no or extremely small effects for the majority of the metabolites evaluated. This study further demonstrated that in order to detect the observed minuscule effects as statistically significant, the sample sizes would need to be extremely large, making such a hypothetical study unfeasible.

In principle, tDCS could affect peripheral metabolite levels via two mechanisms: either indirectly through changes in the central nervous system that would modify peripheral neural responses and consequently peripheral metabolism, or directly through central metabolites passing through the BBB. While tDCS does not appear to lead to long-lasting changes in BBB permeability (20), short-lasting functional changes in its permeability could evoke changes in metabolite levels in the peripheral circulation. Our findings did not support a clinically significant presence of such changes, and the above hypotheses remain to be further tested in animal or human models.

Peripheral metabolic effects and serum effects caused by tDCS have previously been investigated in a limited number of studies.(10–13, 21–23) In these studies, one session of tDCS was found to cause changes in glucose metabolism (10, 21) and cortisol secretion (11–13, 23) when investigating healthy individuals. The sample sizes of the glucose studies were 15 (sham-controlled cross-over design), and 9 (within-subjects design, no sham, measurements before and after stimulation), respectively, while the sample sizes of the cortisol studies ranged from 20 to 60. Interestingly, one of the studies indicated that the hormone-related effect of tDCS varies based on pre-existing conditions. Subjects with high math anxiety experienced reduced serum cortisol with tDCS compared to sham stimulation, while receiving tDCS was associated with no change in serum cortisol in subjects with low math anxiety (13).

In addition, Khedr et al. (22), in a study with 40 participants, observed that 10 consecutive sessions of tDCS lowered pain, a finding that correlated with an increase in serum endorphin levels during the 10-session intervention. Brunoni et al. (24–26) investigated the effect of tDCS on circulating neurotrophins and their receptors (brain-derived neurotrophic factor (BDNF), neurotrophins 3 and 4 (NT-3 and NT-4), nerve growth factor, glial cell line derived neurotrophic factor (GDNF), and soluble tumour necrosis factor receptors 1 and 2), but found no difference between individuals receiving tDCS vs. sham stimulation. A few year later (27), the same group expanded their research to interleukins and tumour necrosis factor alpha, and again observed no differences observed between participants receiving tDCS vs. sham stimulation. In contrast, in a study by Hadoush et al. (28), belonging to the tDCS group vs. sham stimulation was associated with an increase in serum BDNF levels in Parkinson’s disease patients undergoing a 10-session tDCS intervention. The effects of tDCS on the concentrations of soluble neuronal cell adhesion molecules in minimally conscious subjects have also been studied; the authors observed no significant tDCS-related changes (29). Unfortunately, our metabolomics panel did not include any of the aforementioned metabolites, which limits comparisons with these previous observations. However, these studies (with the exception of glucose studies) focused more on larger molecules in peripheral blood in contrast to the small metabolic products we measured.

Some earlier reports have suggested that tDCS may cause intracerebral changes in the concentrations of certain metabolites. For example, Dickler et al. (30) found an increase in dorsolateral prefrontal cortical GABA levels under the stimulated area in patients with a gambling disorder, while Hone-Blanchet et al. (31) recorded elevated striatal levels of N-acetylaspartate and Glx (a combined measurement of glutamate and glutamine), but no differences in the levels of GABA in healthy subjects. Although a previous study (10) did claim that tDCS-induced alterations in central metabolism are reflected in the peripheral circulation, we observed no tDCS-related alterations in the serum levels of N-acetylaspartate or GABA among healthy individuals. Nevertheless, the possibility of such alterations at the cerebral level cannot be ruled out; tDCS may have induced central nervous system alterations that are not reflected as meaningful alterations at the peripheral level.

The main strengths of our study are the relatively large sample size (previous studies focusing on tDCS-induced possible peripheral metabolic changes have utilised samples ranging from 14 to 60 individuals) (13, 32) and the implementation of a randomized, double-blind, controlled study design. Furthermore, in order to reduce potential confounding due to individual lifestyle factors, we provided the study participants with detailed instructions regarding lifestyle behaviours potentially modifying the effects of tDCS before each tDCS/sham stimulation session. All participants were instructed to fast before providing blood samples. Controlling for these factors most likely contributed to the more homogeneous findings between the two study groups, whereas some previous studies have not reported whether they provided similar instructions to their participants.

Some issues need to be taken into consideration while interpreting the findings from our have received a similar tDCS regime (i.e., a 20-minute stimulation session with a current of 2 mA for five consecutive days). While the montage we used is common in both clinical and experimental tDCS studies, caution is advised when extrapolating our results to other montages. The tDCS group had a BMI of 26, compared to the BMI of 25.4 in the control group. While the difference between these group means is statistically non-significant, it is of note that a BMI of 26 is considered as overweight, and tDCS has been demonstrated to have different effects in overweight individuals compared to those of normal weight (decreased systemic glucose uptake in response to tDCS in obese individuals compared to normal weight individuals) (33). Thus we cannot completely rule out BMI affecting the results.

In addition, due to the extensive number of comparisons and the stringent alpha levels resulting from correction to the number of these comparisons, we cannot rule out some real effects erroneously appearing as non-significant. The study protocol allowed a maximum time interval of 5 min between the end of stimulation and the venipuncture, but unfortunately we did not use a timer to record this interval. In practice, the circa 20-metre transfer from the stimulation room to the blood sampling station, as well as preparations necessary for the blood draw, were estimated to take approximately 3 min, leaving approximately 2 min for individual variation in the interval. Therefore, we cannot exclude potential random variation that this time interval may have induced in our findings.

It is also important to note that any tDCS-related changes may have a ceiling effect in the healthy brain. For example, a greater improvement in cognitive task performance following tDCS was observed in neuropsychiatric patients than in healthy controls (34). Therefore, our observations may have been different among, for example, patients with depression. Nevertheless, we sought to provide a healthy male volunteer ‘baseline’ of findings that we hope both our group and others will in the future be able to use as a healthy individual reference when conducting further investigations among patients.

In the present investigation, both the participants and the experimenters were able to distinguish sham and active stimulation at the beginning of the protocol, which is in contrast to some previous studies (35). This might be a result of the different protocols applied here, with its higher currents and greater current density as compared to earlier investigations. In addition, we also had larger study groups, resulting in a higher power to detect smaller failures in blinding. However, this issue should not have significantly impacted on our findings.

Our power calculations suggested that enormous sample sizes would be necessary to detect any changes in most of the studied compounds. However, while the power calculations indicated that some of the metabolites would require sample sizes at the level of thousands of individuals to be detected as statistically significant, some metabolites, such as xanthine and hippuric acid, could be detected as significant with group sizes of less than 100. These power calculations may be beneficial when planning future studies. Furthermore, GABA and glutamate, for which our observations suggest sufficient power for detection with sample sizes of 35 and 164 per group, respectively, are of special interest for clinical tDCS research. Both GABAergic and glutaminergic systems have been suggested to play a significant part in, for example, the pathophysiology of depression (36), and any changes in these markers may be of interest from the point of view of treatment mechanisms or the prediction of treatment efficacy.

## Conclusions

We found that five daily sessions of tDCS, applied to healthy male adults, did not result in significant changes in peripheral blood metabolites. These results indicate that tDCS may be metabolically safe, at least for healthy participants, but more research is needed to determine whether the results would be the same in populations with metabolic disturbances. Further studies with larger samples as well as with female volunteers are also warranted. Our power calculations offer a useful, evidence-based baseline for designing such studies.

## Data Availability Statement

The informed consent received from the participants, as well as the ethics permission from the research ethics committee who reviewed the study protocol, only permits data transfer to countries with data privacy laws compatible with the respective laws in Finland. Therefore, the data cannot be published in an open repository. Nevertheless, the data will be made available on request and can be obtained by contacting the corresponding author (soili.lehto@helsinki.fi).

## Ethics Statement

The studies involving human participants were reviewed and approved by Ethics Committee, Hospital District of Northern Savo, Kuopio University Hospital. The participants provided their written informed consent to participate in this study.

## Author Contributions

Data acquisition—LK, VV. Study design—TK, SL, JW. Data analysis and interpretation—AK, AO-A, A-HJ, TT, TA-S. Project supervision—SL, JW. Wrote the manuscript—AK. Commented on the manuscript—AO-A, A-HJ, TT, TA-S, TK, JW, LK, JN, VV, SL.

## Funding

This study was supported by the Signe and Ane Gyllenberg Foundation, the Finnish Medical Foundation, and VTR research funding. SL was supported by a grant from the Finnish Medical Foundation. AK was supported by Emil Aaltonen Foundation, Finnish Medical Foundation and Jalmari and Rauha Ahonen Foundation. None of the funding sources had any involvement in the study design or execution.

## Conflict of Interest

The authors declare that the research was conducted in the absence of any commercial or financial relationships that could be construed as a potential conflict of interest.
