# Supplementary material for: Anodal tDCS Over the Left Prefrontal Cortex Does Not Cause Clinically Significant Changes in Circulating Metabolites
Source: Front Psychiatry. 2020 May 7;11:403. doi: 10.3389/fpsyt.2020.00403 (PMC7221177; doi:10.3389/fpsyt.2020.00403)
Supplement: Supplementary file 1 [file DataSheet_1.docx]

**Supplementary Methods**

*Statistical data analyses (extended)*

Preliminary inspections of the metabolomic data allowed detecting a total of 2124 missing observations (8.786% of the 24174 values in the dataset). Because excessive amount of missing values within a compound could potentially introduce unanticipated biases, metabolites with ≥50% of missing values within either of the experimental groups were excluded from any further statistical test. Accordingly, 9 metabolites did not pass the selected cut-off and were excluded from further analyses. The remaining 93 metabolites exceeding the cut-off were included in further analyses (see Supplementary Table 1 and Figure 1).

Subsequently, as some of the raw (cleaned) metabolite variables showed skewed distributions, they were transformed to Gaussian distribution where necessary. For each metabolite variable, the raw, negative reciprocal, power 2, power 3, power 4, power 1/2, power 1/3, and natural log transformed variables were tested for deviation from the standard Gaussian distribution using Shapiro-Wilk test. The transformed data showing the smallest deviation from the standard Gaussian distribution were chosen to be finally standardized to the baseline mean and standard deviation. This data pre-processing brought all the metabolites to a similar, more symmetrical distribution (which helped to avoid having non-normal residuals in the later models), stabilized the variance across the entire ranges of concentration, and minimized the influence of extreme outliers, so that the subsequent analyses better could reflect the central tendency of the data.

The main analyses of this study, aiming to investigate the group differences in the longitudinal variation of each metabolite, were implemented through Generalized Estimating Equations (GEEs) models [1], an extension of the Generalized Linear Models (GLM) framework. The GEE models take into account the correlated nature of longitudinal data to estimate appropriate regression coefficients for main effects and interactions. In brief, this GEE modelling started by fitting a GLM model with the following additive form:${g(Y}_{it})=\beta_{0}+\beta_{1}\cdot{Time}_{it}+\beta_{2}\cdot{Group}_{i}+\beta_{3}\cdot{Time}_{it}\cdot{Group}_{i} +e_{it}$. In this equation $g(Y_{it})$ refers to the value for the *i*th individual at time *t* (t=0,1,5) and g(·) being the link function (in this case the identity function, with a Gaussian probability distribution for the metabolite data); $\beta_{X}$ refers to the regression coefficient for each predictor, with $\beta_{0}$ denoting the mean metabolite level at the study baseline, $\beta_{1}\cdot{Time}_{it}$ denoting the metabolite rate-of-change per treatment session, $\beta_{2}\cdot{Group}_{i}$ denoting the differences between groups in the metabolite level at the study baseline, and $\beta_{3}\cdot{Time}_{it}\cdot{Group}_{i}$ signifying the group differences in the metabolite rate-of-change (that is, potential differences in the metabolite changes across time due to the administration of transcranial direct current stimulation respect to sham stimulation); and $e_{it}$ is the error in prediction for each individual and time.

Due to the longitudinal nature of our data, the variances estimated in this preliminary additive model were then multiplied against a working correlation matrix to correct for within-individual correlation. This correlation matrix is normally estimated by the GEE model in a form that matches the expected correlation structure within each individual. Although specifying an appropriate form of the correlation of responses increases the efficiency of these estimates, the model is robust to misspecification of this structure, and therefore, the efficiency gains from exact specification of the structure are considered minimal [2]. The working correlation was specified as “unstructured” for all metabolite models, as it is often considered the most effective in situations of unbalanced longitudinal data with relatively few time points. The preliminary model output is then entered in an iterative reweighted least squares process aiming to minimize the deviation of the parameter estimated respect a perfectly fitted model until they ultimately stabilize and can be consider final.

All GEEs models were developed utilizing the R package “geepack” [3, 4]. The assumptions of GEE (e.g. linearity in the link function, normal distribution and scattering of residuals, heteroscedasticity, etc.) were evaluated and they were found to be reasonably valid.

Due to the complexity of these analyses, we set a p-value threshold for statistical significance in this study that took into account family-wise error rate (Bonferroni method [5, 6]) as well as the intrinsic highly correlated nature of the metabolite data. This was done through dividing the traditional alpha level of significance (0.05) by the maximum number of independent tests, which was determined itself as the product of the minimum number of orthogonal linear (principal) components explaining >95% of the observed variance in the entire metabolics panel at baseline (31 components, Supplementary Figure 2), and the number of model contrasts evaluated (3 contrasts). Accordingly, there were 93 independent tests, thus setting the Bonferroni corrected p-value threshold for statistical significance in this study at p≤5.376e-04.

These main statistical analyses were supplemented with a set of power-related computations utilizing statistical simulations within a sophisticated computer cluster environment. Firstly, we evaluated the sensitivity conferred by the current sample to detect significant “$Time\cdot Group$” coefficients with ≥80% power (for 0.05 and 5.376e-04 type-I error rates). This process was done through simulating 10000 datasets with the current sample size in the way that when modeled as in the present study they offered a pre-specified “time x group” coefficient (values between 0.0001 and 0.25, with increments of 0.0001), while the other coefficients in the model were close to zero and non-significant. The resulting power of detection for that particular coefficient value was noted as the outcome.

Subsequently, for each metabolite in this study, we evaluated the sample size required to detect the corresponding “$Time\cdot Group$” coefficient as statistically significant with ≥80% power (type-1 error rates 0.05 and 5.376e-04). For each metabolite coefficient value, the process started by simulating 10000 datasets including 10 individuals per group in the way that when modeled as in the present study they offered the pre-specified “time x group” coefficient (while the other coefficients in the model were close to zero and non-significant). The power to detect the pre-defined “$Time\cdot Group$” coefficient was then estimated, and if it was <80% then a new series of 10000 datasets were simulated with similar characteristics but adding of either 1 or 25 more individual per group, for evaluations with 0.05 or 5.376e-04 type-I error rate, correspondingly. This incremental process was automatically repeated until the statistical power from the newly created 10000 simulated dataset to detect the pre-specified effect size was ≥80%. The final number of individuals per group for each metabolite coefficient and each type-I error rate was then noted as the outcome of interest. Importantly, metabolites with “$Time\cdot Group$” coefficients <0.005 were excluded from estimations with type-I error rate 0.05; while metabolites with “$Time\cdot Group$” coefficients <0.01 were excluded from estimations with type-I error rate 5.376e-04. This was done to maintain efficiency and avoid excessive computational time, while considering that the number of individuals per group required to detect those small coefficients would be exceptionally large, and therefore difficult to reach in practice in future experimental settings.

**References**

[1] Zeger SL, Liang KY, Albert PS. Models for longitudinal data: a generalized estimating equation approach. Biometrics 1988;44:1049-1060.

[2] Liang KY, Zeger SL. Longitudinal Data-Analysis Using Generalized Linear-Models. Biometrika 1986;73:13-22.

[3] Halekoh U, Hojsgaard S, Yan J. The R Package geepack for Generalized Estimating Equations. J Stat Softw 2006;15:1-11.

[4] Grund S, Robitzsch A, Luedtke O. mitml: Tools for Multiple Imputation in Multilevel Modeling. 2016 [cited; Available from: <https://CRAN.R-project.org/package=mitml>

[5] Armstrong RA. When to use the Bonferroni correction. Ophthal Physl Opt 2014;34:502-508.

[6] Neyman J, Pearson ES. On the use and interpretation of certain test criteria for purposes of statistical inference. Biometrika 1928;20A:66.
