# Supplementary material for: Anodal tDCS Over the Left Prefrontal Cortex Does Not Cause Clinically Significant Changes in Circulating Metabolites
Source: Front Psychiatry. 2020 May 7;11:403. doi: 10.3389/fpsyt.2020.00403 (PMC7221177; doi:10.3389/fpsyt.2020.00403)
Supplement: Supplementary file 2 [file Table_1.docx]

**Supplementary Table 1:** Metabolites excluded because of excessive missing data

| Metabolite | Class | % Missing  Sham/Treatment |
| --- | --- | --- |
| 5-Hydroxy-L-tryptophan | Alpha amino acids and derivatives | 100% / 100% |
| 5-Hydroxyindoleacetic acid | Indoles and derivatives | 100% / 100% |
| Neopterin | Pteridines and derivatives | 100% / 100% |
| Pyridoxine | Pyridines and derivatives | 100% / 100% |
| Cyclic GMP | Purines and derivatives | 100% / 97.5% |
| Nicotinic acid | Pyridines and derivatives | 99.1% / 99.2% |
| Uridine diphosphate glucose | Pyrimidines and derivatives | 95.7% / 95% |
| Glutathione | Alpha amino acids and derivatives | 92.3% / 87.5% |

**Supplementary Table 2:** Results from estimating the sample size required to detect statistically significant value for the “time x group” coefficient found in this study for each metabolite as with 80% power (type-I error rates: 0.05 and 5.376e-04).

| Metabolite | Effect Size Detected  (Time x Group Coefficient) | N per Group  (Type-I Error: 0.05) | N per Group  (Type-I Error: 5.376e-04) |
| --- | --- | --- | --- |
| 2-Aminoadipic acid | 0.073 | 72 | 168 |
| 2-Aminoisobutyric acid | -0.172 | 13 | 31 |
| 3-Hydroxyanthranilic acid | -0.012 | 2650 | 7000 |
| 4-Pyridoxic acid | -0.03 | 420 | 988 |
| Acetoacetic acid | -0.031 | 388 | 918 |
| Adenine | 0.029 | 446 | 1075 |
| Adenosine | -0.061 | 100 | 234 |
| Adenosine monophosphate | 0.005 | - | - |
| Allantoin | -0.049 | 155 | 372 |
| alpha-D-glucuronic acid | 0.032 | 364 | 867 |
| Asymmetric dimethylarginine | 0.012 | 2650 | 7000 |
| Betaine | 0.003 | - | - |
| Carnosine | -0.028 | 480 | 1150 |
| Chenodeoxycholic acid | -0.061 | 103 | 238 |
| Cholic acid | -0.004 | - | - |
| Choline | -0.038 | 258 | 596 |
| Citrulline | -0.036 | 290 | 675 |
| Creatine | -0.072 | 72 | 171 |
| Creatinine | 0.008 | 5975 | - |
| Cyclic AMP | -0.044 | 189 | 453 |
| Cytidine | -0.04 | 233 | 544 |
| Cytosine | -0.012 | 2450 | 7000 |
| D-Ribose-5-phosphate | -0.002 | - | - |
| Decanoylcarnitine | -0.069 | 78 | 183 |
| Deoxycytidine | -0.051 | 143 | 339 |
| Deoxyuridine | -0.018 | 1200 | 2775 |
| Dimethylglycine | 0.053 | 129 | 307 |
| Folic acid | -0.006 | 11100 | - |
| Gamma-Aminobutyric acid | 0.105 | 35 | 80 |
| Gamma-Glutamylcysteine | 0.042 | 210 | 503 |
| Glyceraldehyde | 5.33E-04 | - | - |
| Glycine | 0.033 | 341 | 804 |
| Glycocholic acid | -0.039 | 242 | 565 |
| Guanidoacetic acid | 0.025 | 622 | 1450 |
| Guanosine | -0.026 | 575 | 1400 |
| Hexanoylcarnitine | -0.002 | - | - |
| Hippuric acid | 0.083 | 55 | 128 |
| Homocysteine | -0.029 | 436 | 1050 |
| Homogentisic acid | -0.066 | 87 | 201 |
| Hydroxykynurenine | -0.069 | 78 | 180 |
| Hypoxanthine | -0.02 | 921 | 2200 |
| Inosine | 0.028 | 489 | 1125 |
| Inosinic acid | 0.032 | 365 | 870 |
| Isobutyryl-L-carnitine | -0.042 | 202 | 491 |
| Isovalerylcarnitine | -0.059 | 112 | 257 |
| Kynurenic acid | 0.011 | 3225 | 7500 |
| L-Acetylcarnitine | -0.009 | 4850 | - |
| L-Alanine | 0.029 | 425 | 1025 |
| L-Arginine | 0.016 | 1525 | 3650 |
| L-Asparagine | 0.046 | 176 | 408 |
| L-Aspartic acid | 0.025 | 613 | 1450 |
| L-Carnitine | -0.049 | 154 | 366 |
| L-Cystathionine | -0.039 | 244 | 565 |
| L-Glutamic acid | -0.047 | 164 | 394 |
| L-Glutamine | 0.088 | 50 | 112 |
| L-Histidine | 0.004 | - | - |
| L-Homoserine | 0.072 | 73 | 170 |
| L-Isoleucine | -0.02 | 952 | 2500 |
| L-Kynurenine | 0.024 | 667 | 1550 |
| L-Leucine | -0.03 | 421 | 995 |
| L-Lysine | -0.053 | 137 | 323 |
| L-Methionine | 0.012 | 2550 | 7000 |
| L-Octanoylcarnitine | -0.037 | 220 | 450 |
| L-Ornithine | -0.019 | 1075 | 2525 |
| L-Phenylalanine | 0.013 | 2150 | 7000 |
| L-Proline | -0.05 | 149 | 350 |
| L-Serine | -0.016 | 1525 | 3600 |
| L-Threonine | 0.026 | 561 | 1325 |
| L-Tryptophan | 0.036 | 285 | 692 |
| L-Tyrosine | -0.045 | 179 | 430 |
| L-Valine | -0.04 | 233 | 547 |
| Myo-inositol | 0.031 | 391 | 937 |
| N(tele)-methylhistamine | 0.032 | 364 | 869 |
| NAD | 0.015 | 1600 | 3775 |
| Niacinamide | -0.003 | - | - |
| Normetanephrine | -0.032 | 359 | 841 |
| O-Phosphoethanolamine | -0.029 | 434 | 1050 |
| Orotic acid | 0.019 | 1025 | 2500 |
| Pantothenic acid | 0.023 | 714 | 1675 |
| Propionylcarnitine | -0.085 | 51 | 124 |
| Sorbitol | 0.036 | 278 | 674 |
| Spermidine | 0.045 | 188 | 443 |
| Succinic acid | -0.014 | 1900 | 7000 |
| Sucrose | 0.004 | - | - |
| Symmetric dimethylarginine | 0.06 | 103 | 244 |
| Taurine | -0.039 | 239 | 562 |
| Taurochenodesoxycholic acid | -0.059 | 112 | 257 |
| Taurocholic acid | 0.005 | - | - |
| trans-4-Hydroxy-L-proline | -0.032 | 359 | 842 |
| Trimethylamine N-oxide | -0.037 | 272 | 635 |
| Uracil | 0.03 | 395 | 942 |
| Xanthine | 0.103 | 36 | 83 |
| Xanthosine | -6.27E-04 | - | - |
