# Supplementary material for: Anodal tDCS Over the Left Prefrontal Cortex Does Not Cause Clinically Significant Changes in Circulating Metabolites
Source: Front Psychiatry. 2020 May 7;11:403. doi: 10.3389/fpsyt.2020.00403 (PMC7221177; doi:10.3389/fpsyt.2020.00403)
Supplement: Supplementary file 3 [file Image_1.pdf]

Supplementary Figure 1: Missing value information

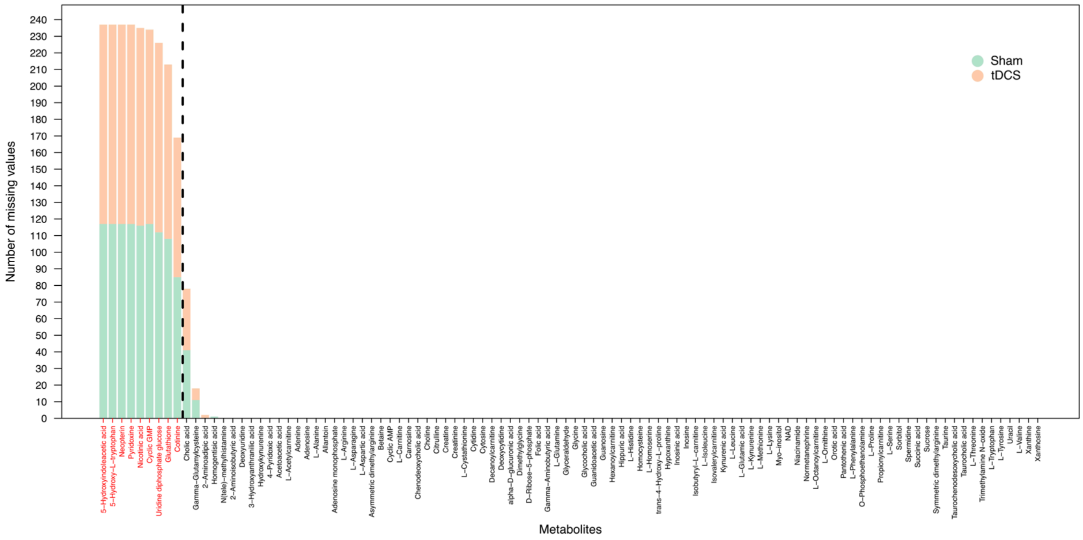

Note: The bars represent number of missing values (vertical axes) for each metabolite (horizontal axes) and group (Sham in green, while tDCS in orange). The names of the metabolites excluded from the analyses according to the criterion specified are in red colour.

Supplementary Figure 2: Pareto chart from principal components analyses of the metabolite data.

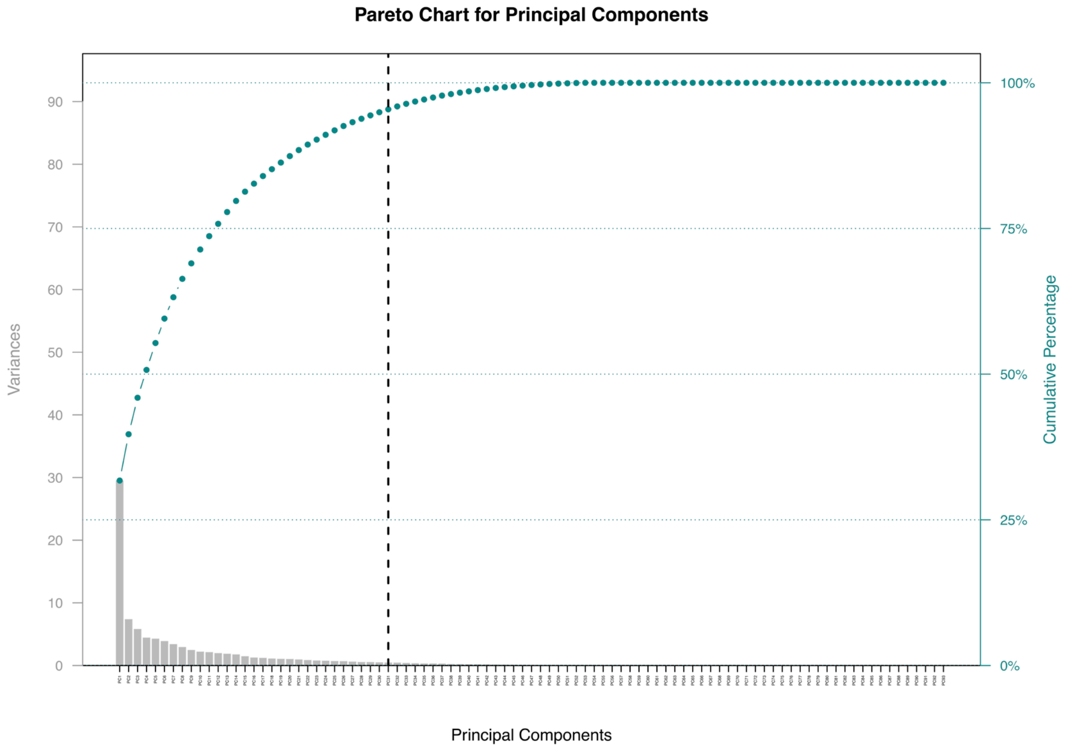

Note: the dashed vertical line indicates the number of principal components explaining ≥95% of the variance within the metabolite data analysed.
